# Reduced neutralization of SARS-CoV-2 Omicron variant by BNT162b2 vaccinees’ sera: a preliminary evaluation

**DOI:** 10.1080/22221751.2022.2045878

**Published:** 2022-03-10

**Authors:** D. Mileto, V. Micheli, C. Fenizia, M. Cutrera, G. Gagliardi, A. Mancon, F. Bracchitta, A. De Silvestri, G. Rizzardini, A. Lombardi, M. Biasin, M. R. Gismondo

**Affiliations:** a Laboratory of Clinical Microbiology, Virology and Bioemergencies, ASST Fatebenefratelli Sacco, L. Sacco University Hospital, Milan, Italy; b Department of Biomedical and Clinical Sciences L. Sacco, University of Milan, Milan, Italy; c Department of Pathophysiology and Transplantation, University of Milan, Milan, Italy; d Clinical Epidemiology and Biometeric Unit, Fondazione IRCCS Policlinico San Matteo, Pavia, Italy; e Division of Infectious Diseases, ASST Fatebenefratelli Sacco, L. Sacco University Hospital, Milan, Italy

Mutations in viral genome arise as a natural by-product of viral replication and their fate is determined by natural selection. Accordingly, in the context of “variants of concern” (VOC), SARS-CoV-2 evolution has been characterized by wide variation, altering transmissibility, antigenicity, immune response and disease severity [1]. The most recent Omicron variant (B.1.1.529) puts scientists on alert because of its Spike protein extended mutational pattern, which could challenge the effectiveness of the worldwide vaccinal campaign: the receptor-binding domain (RBD) presents 15 amino acid changes, some of which have been associated with increased binding affinity to ACE2; in addition, three mutations close to S1/S2 furin cleavage site may increase transmissibility, while the presence of several deletions and insertions in the N-terminal domain (NTD) has been shown to compromise some established PCR assays [1].

Vaccines are considered to be the best available solution for controlling and limiting the ongoing COVID-19 pandemic [Rella] [2]. Taking into account that nearly 61.9% has received at least one dose of a COVID-19 vaccine [ourworld] (https://ourworldindata.org/covid-vaccinations?country=OWID_WRL), herein we verified if humoral immunity elicited by these vaccines is able to neutralize the Omicron strain, as already demonstrated for other VOCs [3]. To this end, serum samples from 37 immunocompetent health care workers (HCWs), collected 1 month after the administration of the BNT162b2 vaccine second dose, were assessed for their neutralizing activity (NA) in a virus neutralization assay (NTA) against SARS-CoV-2 first Italian Omicron strain (accession number: EPI_ISL_6777160) [4]. NA results were compared with those obtained against lineage B.1 (EU) (accession number: EPI_ISL_412973) and lineage B.1.617.2 (Delta) (accession number: EPI_ISL_1970729) lineages in the same cohort [3].

We observed reduced NA of BNT162b2 vaccinees’ sera against the Omicron variant (Figure 1(A)): a 7.3-fold (I.C.95%:5.65–9.34) and a 3.15-fold (I.C.95%:2.52–3.94) reduction was detected if compared with EU (Figure 1(C)) and Delta (Figure 1(B)), respectively (*p* < 0.0001). In particular, considering as non-protective a neutralizing antibodies titre of 1:10 and as positive and protective a ≥ 1:20 one, a total of 24/37 (64.8%) subjects resulted protected, further 12/37 (32.4%) showed a positive non-protective concentration and in only one case (2.7%) a negative result was observed [5]. Analyses of correlation between antibody titres measured with CLIA assay (LIAISON® SARS-CoV-2 TrimericS IgG) and NTA performed on SARS-CoV-2 Omicron variant upon the second dose revealed a significant positive correlation (Figure 1(D); *p* = 0.0267). Noteworthy, three weeks after a third dose both antibody titres (75% increase; I.C.95%:45–105%) (Figure 1(E)) and NA (3.03-fold; I.C.95%:1.89–4.86) (Figure 1(F)) increased (n = 5), enhancing the immune protection.

**Figure 1.**
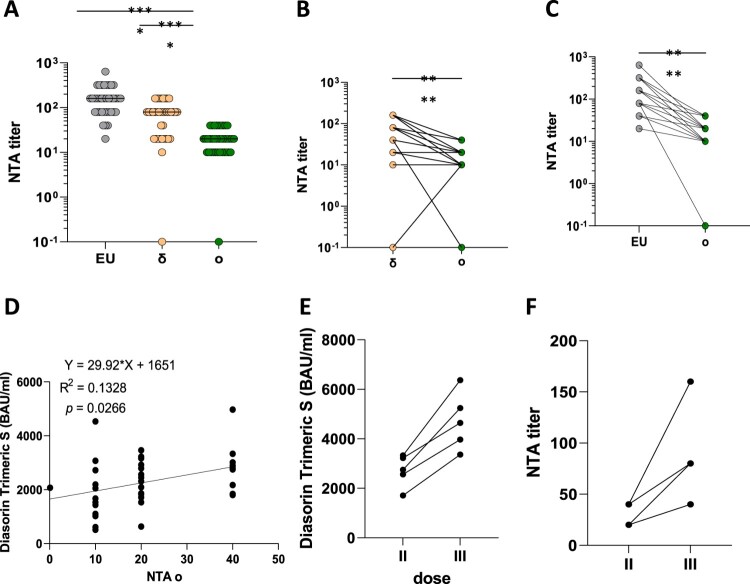
SARS-CoV-2 variants of concern (VOC). Panel (A) Neutralization assay (NTA) performed on the SARS-CoV-2 lineage B.1 (EU) and 2 VOCs, d and o. Panels B and C comparison between the Omicron variant and the EU and delta variant, respectively. Lines connect the NTAs of each individual subject. Panel D shows the correlation between anti-SARS-CoV-2 specific antibodies and neutralization assays (NTA) measured 1 month post two doses of the BNT162b2 vaccine. Three weeks after a third dose both antibody titres (Panel E) and neutralizing activity (NA) (Panel F) increased. **** = *p* <0.0001.

As we and others [6–8] observed an *in vitro* reduced NA against Omicron compared to other variant*,* we can speculate that this might be translated into a higher susceptibility to breakthrough infection or reinfection in SARS-CoV-2 vaccinees. However, we also noticed that *in vitro* the isolated Omicron clone had a slower propagation rate and a milder cytopathic effect compared with the previous variants, confirming data from Basile et al. [9]. This observation could justify the fact that, so far, no clinical evidence of a more severe progression following Omicron infection has been documented.

Notably, we also reported a positive correlation between the quantity of antibodies detected by CLIA assay and NA against the Omicron variant tested by NTA. This may suggest that antibody quantification by sierological test may be used as a surrogate marker of their neutralizing efficacy.

Despite the limited sample number, the serial sera samples from five participants collected pre-and post-booster demonstrate that the third dose of the BNT162b2 vaccine increased Omicron neutralization titres and may substantially reduce the risk of after vaccine symptomatic infections. However, to date, the kinetic of their concentration over time is undefined. Hence, antibody titre monitoring is mandatory to verify if a boost dose is sufficient to promote antibody affinity maturation and their long-term maintenance or if newer generation vaccines should be designed to cover this variant. T cell-mediated immunity should be assessed as well: while several RBD mutations of Omicron variant’s spike protein result in an immunological escape from antibody-mediated protection, T cell immune responses towards non-surface proteins following infection or vaccination seems to be still effective against Omicron [10].

Overall, our findings suggest that the SARS-CoV-2 Omicron variant can be neutralized by nearly 60% of sera collected from BNT162b2 recipients and booster vaccine doses are necessary to curtail the risk of SARS-CoV-2 transmission, mainly in those subjects displaying waning immunity post full vaccination.

## Data Availability

The data that support the findings of this study are available from the corresponding author, D.M., upon reasonable request.
